# Unraveling Financial Exploitation in Older Adults: A Multidimensional Approach

**DOI:** 10.1093/geroni/igaf122.103

**Published:** 2025-12-31

**Authors:** Gali Weissberger, Yoav Bergman, Duke Han

## Abstract

A growing body of work has identified the multifaceted nature of the risk factors and consequences of financial exploitation (FE) in older adults, demonstrating the importance of psychosocial, cultural, and cognitive factors. While investigating each of these domains individually is crucial to understanding the phenomenon, considering them together is rarely done. The goal of this symposium is to present novel findings which integrate cognitive, cultural, and psychosocial risk factors of FE. From a cognitive perspective, Sunderaramen introduces a new online money management task, and demonstrates a relationship between auditory memory and identification of erroneous transactions, a skill necessary for avoiding credit card fraud and other forms of FE. Moreover, Hoffman demonstrates the interactive links between associative memory, quality of life, and FE vulnerability. From a sociocultural perspective, Noriega-Makarskyy elaborates upon the relationship between FE vulnerability and social relationships to show that relationship depth, and specifically a strong sense of belonging, is associated with less FE vulnerability, while having distinct social roles is not, and Weissberger explores how culture may modify associations between experienced FE and psychosocial factors. Finally, Bergman employs a longitudinal design to examine the cross-lagged association between subjective age and FE vulnerability. Together, these presentations provide a nuanced understanding of FE, emphasizing cognition, culture, age-related perceptions, and social aspects which mitigate FE risks and consequences. The discussion (led by D. Han) will integrate the implications gleaned from each individual presentation and highlight the importance of taking a multifaceted approach to evaluating FE risks and consequences.

